# The economics of implementing Magnet^®^ designation: relevant parameters for a cost-benefit analysis in the German hospital context

**DOI:** 10.1186/s12913-026-15346-9

**Published:** 2026-08-12

**Authors:** Carolin Gurisch, Julia Köppen, Claudia B. Maier, Joan Kleine, Reinhard Busse

**Affiliations:** 1 Department of Healthcare Management, Technische Universität Berlin, Berlin, Germany; 2 Department of Health Sciences, BQS Institut für Qualität und Patientensicherheit GmbH, Hamburg, Germany; 3 School of Public Health, Bielefeld University, Bielefeld, Germany

**Keywords:** Hospitals, Magnet, Nursing, Quality of health care, Cost-benefit analysis, Implementation science

## Abstract

**Background:**

The U.S. Magnet^®^ model is an organizational intervention that aims to improve quality of care and patient outcomes by improving the hospital work environment for nurses. Its financial impact on German hospitals remains unclear. This study aimed to identify parameters for conducting a cost-benefit analysis for German hospitals seeking Magnet^®^ designation and assess availability of relevant data.

**Method:**

The hospital organizational perspective was adopted. A scoping review was conducted in January 2024 using Medline, CINAHL, Web of Science Social Sciences Citation Index and Cochrane Reviews to identify potential costs and cost-saving opportunities for hospitals implementing Magnet^®^. Interviews with six key informants from nursing management from five German hospitals, participating in the Magnet4Europe study, were conducted in September and October 2024 to contextualize these findings for German hospitals. Market prices for each cost-saving potential were researched; further parameters to conduct a cost-benefit analysis were specified. The reporting of the economic components of the study adhered to the consolidated health economic evaluation reporting standards.

**Results:**

Potential costs include (i) fees of the American Nurses Credentialing Center, (ii) fees for benchmarking, (iii) expenses for professional development and staff and (iv) expenses for supporting resources, data management and innovations. In contrast to existing economic studies, German hospitals underlined potential expenses related to nurses’ academization as part of nurses’ professional development, and data management of nurse-sensitive indicators. Possible cost savings were identified from improvements in (i) nurse-sensitive indicators, (ii) nursing personnel indicators, and (iii) hospital performance. In contrast to existing studies, German hospitals stressed potential cost savings related to hospital performance. The possible cost-savings identified as relevant for the German hospitals were assigned market prices, although no values for injurious falls, pressure injuries category 2–4, and turnover costs were identified for the German healthcare system.

**Conclusion:**

Potential key expenses and cost-saving opportunities for implementing Magnet^®^ designation in hospitals were identified and contextualized for German hospitals. Substantial data gaps currently impede a cost-benefit analysis and underscore critical implementation-relevant evidence gaps that require further research.

**Supplementary Information:**

The online version contains supplementary material available at 10.1186/s12913-026-15346-9.

## Introduction

Hospitals worldwide face a global shortage of healthcare professionals [1, 2]. The healthcare workforce shortage particularly affects nurses, the largest occupational group in hospitals [2]. A shortage of about 2.5 million nurses in hospitals, medical centers and public health facilities is projected across 23 OECD countries, including Germany, for 2030 [3]. Apart from demographic factors, such as nurses leaving the nursing profession due to retirement and a rising demand for nurses due to an ageing population, unattractive working conditions contribute to nurses leaving the profession prematurely and deter potential new recruits [4, 5].

The impact of working conditions on nurses and patient outcomes has been extensively studied. The RN4CAST study, a cross-sectional study conducted in twelve European countries from 2009 to 2011, and demonstrated that nurse outcomes, including job satisfaction, intention to leave, and burnout, were influenced by nurses’ work environment [6]. Patient outcomes, such as mortality and satisfaction, were also associated with the nursing work environment (ibid.). These findings were further supported by a literature review examining the effects of work environments on nurse and patient outcomes [7]. A 2021 cross-sectional study in the United States (U.S.) reinforced these results, showing that inadequate staffing levels and adverse work environments were related to higher turnover, increased burnout, and lower patient safety ratings [8]. A cross-sectional survey examining antecedents of nurses’ job satisfaction in the U.S. found leadership, staffing, interprofessional collaboration, professional practice and development opportunities to be central factors [9]. In Germany, a cross-sectional survey of nurses in acute and long-term care aimed at identifying key factors contributing to the attractiveness of the nursing profession and found that salary, the ability to reconcile family and career, and the opportunity to provide high-quality professional care were essential [10].

The Magnet^®^ Recognition Program is a model of organizational redesign by the American Nurses Credentialing Center (ANCC) [11]. It aims to enhance hospital work environments and to promote excellence in nursing, contributing to improved patient safety (ibid.). Its requirements are divided into five central principles: (I) transformational leadership, (II) structural empowerment, (III) exemplary professional practice, (IV) new knowledge, innovation, & improvements, (V) empirical quality results [12]. Implementing the requirements demands building a corresponding culture within the hospital and takes several years (ibid.). As of January 2026, two parts of a Finnish hospital and a Spanish hospital have received Magnet^®^ designation, making them the only Magnet^®^ designated hospitals in Europe and adding to the more than 600 Magnet^®^ designated hospitals in the U.S. and internationally [13].

Many studies have demonstrated benefits of Magnet^®^ hospitals, including better work environments for nurses, resulting in lower levels of burnout and job dissatisfaction, reduced nurse turnover, enhanced quality of care and improved patient outcomes [14–17]. Amid the analyzed benefits of Magnet^®^, the Magnet4Europe study was conducted. The study aimed to support 60 European hospitals from six countries (Belgium, England, Germany, Ireland, Norway, Sweden) in the implementation of the Magnet^®^ model from 2020 to 2024 [18, 19].

When implementing the Magnet^®^ model and pursuing Magnet^®^ designation, hospitals must consider not only the potential benefits but also the associated costs. Different data sources suggest hospitals’ financial struggles. A data analysis from 1,500 hospitals in nine European countries, already published in 2014, found that one in five hospitals had a high probability for default [20]. In Germany, a representative survey of nearly 400 general hospitals in 2023 showed that 54% of the hospital sreported an annual deficit in 2022 and nearly 80% of the hospitals evaluated their financial situation as unsatisfactory [21].

The economic impact of hospitals gaining Magnet^®^ designation, which is relevant for hospital decision-makers, has been explored in some studies by contrasting the costs and financial savings for hospitals. A business case for a hypothetical 500-bed hospital in the U.S. estimated a return-on-investment of approximately $2.3 million per annum for Magnet^®^ designated hospitals [22]. The analysis incorporated estimated savings from improved clinical quality, workforce outcomes, and improved workflow efficiency, as well as implementation costs, including Magnet^®^ fees and expenses for document preparation (ibid.). Building on the assumptions proposed by Drenkard et al., a subsequent study developed a business case for hospitals with fewer than 100 beds by adjusting both cost and savings estimates to the context of smaller hospitals [23]. The analysis incorporated improvements in quality and workforce outcomes as well as costs related to Magnet^®^ fees, document preparation, benchmarking of nurse-sensitive indicators, a Magnet^®^ program director, and participation in Magnet^®^ conferences, resulting in projected annual savings of approximately $708,000 for Magnet^®^ hospitals (ibid.). The research focus was expanded by an international business case for a 360-bed hospital in Saudi Arabia that achieved Magnet^®^ designation [24]. Based on hospital-specific data, the study estimated savings of approximately $2.3 million over five years, considering both quality- and workforce-related benefits, and returns on nurse-led innovations as well as implementation costs, including Magnet^®^ fees, benchmarking, consultants, conferences, and nurses’ certification costs (ibid.). For the monetization of cost savings, estimates from the U.S. were used (ibid.). In a further study, a framework for hypothetical hospitals with 500 beds in the U.S. was established, displaying areas for cost-saving opportunities, its research evidence and costs of implementing Magnet^®^ [25]. For instance, cost-saving opportunities include improved clinical quality, nurse and patient satisfaction, increased inpatient revenues, higher performance in value-based purchasing initiatives, and returns on nurse-led innovation projects, while costs comprise Magnet^®^ fees (ibid.).

However, no similar studies have been conducted for hospitals in Europe or Germany. Due to the differences between the healthcare systems, this leaves a gap in understanding the financial and strategic impact of gaining Magnet^®^ designation in European or German hospitals. Against this backdrop, this study followed an exploratorye approach. Its aim was to identify and monetize relevant parameters that could inform a cost-benefit analysis from the perspective of German hospitals.

A cost-benefit analysis was chosen because it enables the translation of diverse, interdependent outcomes into a common monetary metric, allowing a comprehensive assessment of the overall value of Magnet^®^ designation for hospitals. Magnet^®^ designation is a multifaceted organizational intervention with effects across multiple dimensions, as demonstrated in the international literature [14–17]. Hence, the cost-benefit approach was considered more appropriate than other forms of health economic evaluation, such as a cost-effectiveness analysis, which requires a single effectiveness measure.

## Method

In order to identify relevant parameters for a cost-benefit analysis of Magnet^®^ designation in German hospitals, a multi-step approach was applied. First, a scoping review of the literature was conducted to identify reported effects of Magnet^®^ designation on patient outcomes, nurse outcomes, hospital performance, and associated costs. Second, expert interviews with representatives from German hospitals implementing the Magnet^®^ model were conducted to contextualize these effects within the German healthcare system. Third, potential cost-saving opportunities were monetized using available market prices and publicly accessible data sources, and further parameters needed for a cost-benefit analysis were researched.

Potential costs and cost-saving opportunities were examined from the hospital perspective, as hospitals are the decision-makers regarding whether to pursue Magnet^®^ designation. The reporting of the economic components of the study adhered to the consolidated health economic evaluation reporting standards (CHEERS) [26].

### Scoping review

First, a scoping review was conducted to identify the reported benefits of Magnet^®^ designation on patient outcomes, nurse outcomes, and hospital performance, as well as the costs associated with implementing the Magnet^®^ model and obtaining Magnet^®^ designation [27].

The present scoping review builds on a previously published scoping review that identified and compared international hospital accreditation and certification schemes, its concepts and their included outcome indicators [28].

The underlying scoping review searched Medline, CINAHL, Web of Science Social Sciences Citation Index and Cochrane Reviews in January 2024 using search terms developed based on the SPICE framework (setting, perspective, intervention, comparison, evaluation) [29]. The search strategy comprised four blocks: (i) hospitals (‘hospital’), (ii) nursing (‘nurse’, ‘nursing’), (iii) quality (‘quality’, ‘improvement’, ‘performance’, ‘safety’, ‘excellence’), and (iv) accreditation and certification schemes (‘accreditation’, ‘certification’, ‘designation’, ‘distinction’). Where available, these free terms were supplemented with controlled terms specific to each database. Accordingly, the search strategy was adapted for each database. The detailed search strategy and full methodological description of the scoping review are reported elsewhere [28].

While the original review examined seven accreditation systems, the present study focused specifically on Magnet^®^ designation. Of the 124 studies, those addressing Magnet^®^ were first identified. In a second step, studies were assessed for eligibility based on predefined inclusion and exclusion criteria. Studies were included if they examined the effects or costs associated with Magnet^®^ designation in hospital settings, regardless of geographical location. As the objective of the present study was to identify parameters relevant for economic evaluation, the focus lay on economic analyses and quantitative studies reporting measurable outcomes that could potentially be monetized. Only studies published in English or German were considered; all other study types and languages were excluded.

Study selection followed a two-stage screening process. First, titles and abstracts were screened for relevance based on the defined inclusion and exclusion criteria by two reviewers (CG, JoK). Second, full-text articles were assessed for eligibility (CG).

Data extraction was performed using a structured data chart developed for this study. Extracted items included: author, year of publication, country, study type, reported outcomes related to costs and their magnitude, reported outcomes related to cost savings and their magnitude associated with Magnet^®^ implementation.

No quality assessment of the studies was conducted, as the focus was on capturing a broad range of evidence on the potential economic effects of Magnet^®^ designation rather than comparing study results.

In addition, the website of the ANCC, which is responsible for awarding Magnet^®^ designation, was reviewed to obtain further information on the costs associated with achieving Magnet^®^ status. Literature reviews were additionally screened to identify further relevant outcomes and references.

### Expert interviews for contextualization

Second, interviews were conducted to contextualize the identified Magnet^®^-related costs and cost-saving potentials within the German healthcare system. Purposive sampling was applied. German hospitals were selected that actively participated in the Magnet4Europe project, ensuring that they had relevant insights into the effects of implementing the Magnet^®^ model. The aim was to interview one to two nurses per hospital who were directly involved in the implementation process and who could provide informed perspectives on the financial implications and the potential cost savings of Magnet^®^ designation for patient outcomes, nurse outcomes, and hospital performance.

In total, five interviews with six representatives from five German hospitals were conducted. Given the exploratory nature of the study and the purposive sampling approach, the sample provided sufficient information power for the aim of identifying and contextualizing relevant parameters for a cost-benefit analysis [30]. Interviewees were repeatedly informed about the voluntary nature of participation and data protection procedures. All interviews were conducted by the same interviewer (CG), who has several years of experience in qualitative interviewing. The interviewer was known to all participants from a previous professional context. The interviews were conducted digitally between September and October 2024, with one interviewee per hospital, except in one case where two interviewees were interviewed together. All interviews were audio-recorded, and all interviewees provided written informed consent in advance.

A semi-structured interview guide was developed based on the findings from the scoping review and encompassed two parts: (i) perceived and anticipated effects of Magnet^®^ designation on patient outcomes, nurse outcomes, hospital performance, and related financial savings; and (ii) current and expected costs of implementing the Magnet^®^ model and obtaining Magnet^®^ designation (appendix A).

Across all interviews, a structured analytical approach was applied. The interview content was systematically mapped to predefined deductive categories derived from the interview guide, which itself was based on the findings from the scoping review. Within these categories, relevant information from the audio recordings was summarized. In addition, new themes emerging from the data were summarized inductively and added as additional categories. A category framework with definitions was developed and iteratively refined to ensure consistent categorization across all interviews. Subsequently, findings on potential costs and cost-saving opportunities were summarized across all interviews in a structured matrix aligned with the deductive and inductive themes. These aggregated interview findings were then compared with the findings of the scoping review to assess their relevance and applicability to the German healthcare context. This comparison was also conducted using a matrix-based approach to enable systematic juxtaposition of interview evidence and international literature findings.

Ethics approval for conducting the expert interviews was granted. The principles of the Declaration of Helsinki were followed.

### Monetization of cost-saving opportunities and identification of additional required parameters

To contrast potential costs and savings, the identified parameters were expressed in monetary units, allowing the results to be presented as a net benefit. For each cost-saving opportunity identified in the literature and expert interviews, available market prices were researched with a focus on German data sources.

To identify relevant cost estimates, the database search was supplemented by targeted web searches of German public bodies, statistical offices and grey literature.

Additionally, further parameters needed for a cost-benefit analysis, as proposed by the framework of Mishan and Quah [31], were assessed with regard to their applicability to the German healthcare system.

## Results

Relevant parameters for conducting a cost-benefit analysis in the German hospital context, following Mishan and Quah, were operationalized through the systematic identification of potential costs and benefits, hence the potential cost-savings, the determination of values for the benefits, the selection of a discount rate, and the choice of a suitable comparison group [31].

### Overview of included studies, potential costs and cost savings

**Fig. 1:**
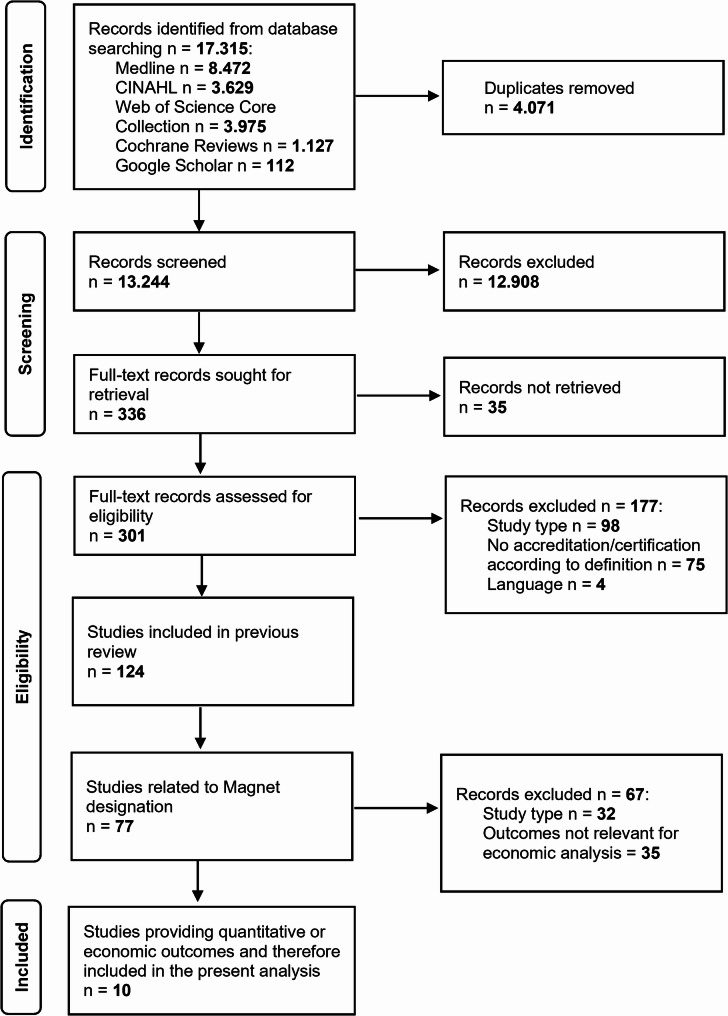
PRISMA-ScR flow diagram

The study selection process of the underlying scoping review is presented in Fig. 1. In total, ten studies were included.

Interviewees included Magnet^®^ directors, members of the nursing directorate, and nursing researchers who had been involved in the implementation of the Magnet^®^ model and who had been familiar with the concept for several years. The participating hospitals were located across different federal states in Germany, covering different geographical regions, and varied in size, ranging from 200 to 1,500 beds.

A comparison of the identified literature on the costs and positive effects of Magnet^®^ designation on patient outcomes, nurse outcomes, and hospital performance with the interview results is provided in appendix B.

The potential costs of implementing Magnet^®^ that were identified as relevant to the German healthcare context encompass four categories: (i) fees by the ANCC, (ii) fees for benchmarking, (iii) hospital’s costs for professional development and staff, (iv) costs for supporting resources, data management, and innovations (Table 1). In contrast, the cost-saving potential is divided into three categories: (i) improved nurse-sensitive indicators, (ii) enhanced nursing personnel figures, and (iii) better hospital performance (Table 1).

**Table 1:** Potential costs and cost-saving opportunities of pursuing Magnet^®^ designation for German hospitals

	Categories	Sub-categories
Potential costs of pursuing Magnet^®^ designation in German hospitals	Fees by the ANCC	Pre-Intent Program subscription
		Magnet^®^ material
		Application fee
		Appraiser fees
		Site-visit costs
	Fees for benchmarking	Benchmarking nurse satisfaction
		Benchmarking patient satisfaction
		Benchmarking nurse-sensitive indicators
	Hospital’s costs for professional development and staff	Professional development of nurses
		Professional development of leadership
		Academization of nurses
		Participation in Magnet^®^ conferences
		Staff (e.g. Magnet^®^ coordinator)
	Costs for supporting resources, data management, and innovations	Implementation costs for data collection, data extraction, data organization
		Access costs to nursing-specific literature
		Costs for the implementation of nurse-led innovations
		Involvement of external consultants
Potential cost savings of pursuing Magnet^®^ designation in German hospitals	Improved nurse-sensitive indicators	Fewer falls with injuries
		Fewer hospital-acquired pressure injuries categories 2–4
	Improved nursing personnel indicators	Lower nurse turnover rate
		Lower use of agency nurses
		Fewer sick days
	Improved hospital performance	Nurse-led innovations
		Extension of nursing services
		Reduction in the length of stay

These categories, the findings on the market prices for the cost-saving opportunities and further parameters for conducting a cost-benefit analysis are explained in the following sections. The magnitude of the costs depends on the individual hospitals’ approaches to implementing the Magnet^®^ model and their sizes. Therefore, the costs’ magnitude will not be addressed further in the following.

### Potential costs of implementing Magnet^®^ in German hospitals

#### **Fees by the ANCC**

There are costs that are incurred in any case by the ANCC and optional costs. The application fee as well as expenses for external verification of compliance with the Magnet^®^ requirements, including appraiser fees and site visit costs, must be considered in every case [32] and were identified by several economic studies as relevant cost components [22–25]. Optional costs include participation fees for the Magnet^®^ Pre-Intent Program [33]. This program is a voluntary option for hospitals that helps them prepare for the Magnet^®^ application process (ibid.) and was included in one economic study analyzing the costs and cost savings when obtaining Magnet^®^ designation [24]. Generally, information about the program’s requirements, summarized in the Magnet^®^ Manual, is subject to fees [34]. However, interviewees reported cases in which this information was provided for free; for example, hospitals participating in the Magnet4Europe project received the manual at no cost. Nevertheless, these potential costs should still be considered and were also included in another economic study [23]. 

#### **Fees for benchmarking**

Hospitals must demonstrate nurse satisfaction, patient satisfaction, and nurse-sensitive indicators exceeding an external national benchmark mean [35]. The external benchmark data need to meet requirements by the ANCC; organizations offering such databases are referred to as vendors [36]. Only one identified study has included all three benchmarking components [23], whereas expenses for benchmarking nurse-sensitive indicators were considered relevant by two studies [23, 24]. All parts of benchmarking costs were considered inherent by the interviewees. In the absence of a national benchmark for inpatient nurse-sensitive indicators, Magnet^®^-applying hospitals outside the U.S. are allowed to benchmark internally, eliminating the need for an external vendor [35], which can reduce costs. 

#### **Hospital’s costs for professional development and staff**

Magnet^®^ requires hospitals to support nurses’ professional development, for example, by fostering accredited training courses or workplace-based courses, and achieve a target number of nurses who have participated in continuing education measures [35]. These expenses are included, following the approach of another economic study [24]. Additionally, interviewees consistently reported that hospitals cover leadership training costs, for example related to the Magnet^®^ principle ‘Transformational Leadership’. Consequently, these expenses are considered relevant though they were not addressed by prior studies. Also, Magnet^®^ mandates that all nursing leadership positions hold at least a baccalaureate degree in nursing at the time of application [35]. Hospitals must also demonstrate how they support nurses in obtaining higher academic qualifications and reaching the required proportion of academically trained nurses (ibid.). Any external costs associated with degree programs funded by the hospital for fulfilling the Magnet^®^ requirement are included, contrasting existing studies that did not take these expenses into account. Additionally, some interviewees indicated that nurses are given paid free time for their studies; such costs are excluded in this study, as they may affect productivity in ways that cannot be accounted for in a cost-benefit analysis. Staff hired or assigned specifically to coordinate the Magnet^®^ implementation as well as expenses related to attending Magnet^®^ conferences [37] were identified as relevant by interviewees and align with the inclusion in prior economic studies [23, 24]. Therefore, these expenses are included. 

#### **Costs for supporting resources**,** data management**,** and innovations**

Interviewees highlighted the engagement of external consultants, for example, to support the implementation of the Magnet^®^ principles or to assist with the preparation of application documents, making these costs an integral part of a cost-benefit analysis, consistent with another economic study [22–25]. Additionally, expenses for nurse-led innovations, as part of the Magnet^®^ component ‘New Knowledge, Innovations, & Improvements’ [12], need to be included. For instance, processing non-stock items in the operating room was considered a nurse-led innovation and included in another economic study [24].

Further expenses were highlighted by the interviewees that were not addressed in previous economic evaluations. First, interviewees reported external implementation costs for data collection, extraction and organization, needed for the benchmarking of nurse-sensitive indicators, which makes these potential costs relevant for the cost-benefit analysis in the German healthcare context. Second, interviewees indicated that not all hospitals have access to nursing literature databases which is necessary to stay informed of the latest research, implementing evidence-based care and contributing to nursing science, as required by the Magnet^®^ principles. This means that these costs need to be taken into account.

### Potential cost savings of implementing Magnet^®^ in German hospitals

#### **Nurse-sensitive indicators**

Hospitals are required to provide data collection on four nurse-sensitive clinical indicators for inpatient units [35]. Data collection on falls with injury is mandatory, while the remaining three indicators are self-selected by each hospital from a predefined list (ibid.). Studies have shown that Magnet^®^ hospitals demonstrate improved performance compared with non-Magnet^®^ hospitals when analyzing nurse-sensitive indicators [17, 38–40].

The potential cost savings associated with declines in injurious fall rates were incorporated in several economic studies [22–25] and its relevance was confirmed by the interviewees. Also, interviewees highlighted the data availability of pressure injuries, stemming from existing data collection mandated by statutory quality assurance requirements, resulting in this indicator beingincluded as well. This also aligns with existing economic studies [22–25]. Data availability for other indicators was reported to be scarce by the interviewees. Consequently, no additional nurse-sensitive indicators are included as cost-saving opportunities, although Magnet^®^ applicants typically have to benchmark a broader set of indicators.

#### **Nursing personnel figures**

Several studies have demonstrated positive effects of Magnet^®^ designation on nursing personnel metrics, such as reduced turnover, fewer sick days and lower reliance on agency nurses [14, 16, 17]. While several economic studies incorporated cost savings from reduced turnover rates [22–25], only one accounted for agency nurse use [22, 25] and none included cost savings from a decrease in sick days. Interviewees confirmed or anticipated the relevance of all three metrics; therefore, these are incorporated as cost-saving opportunities. 

#### **Hospital performance**

Potential cost-saving initiatives within the hospital, led by nurses, were considered in some economic studies [24, 25], their relevance was confirmed by interviewees, and they are therefore considered relevant.

In contrast to existing studies, interviewees also highlighted the potential expansion of nursing services relevant for reimbursement through enhanced nursing roles and skill sets. They further noted the possibility of reducing length of stay through improved patient care resulting from the implementation of the Magnet^®^ principles, along with associated cost savings. Hence, these aspects are also included as cost-saving opportunities.

### Monetization of cost-saving opportunities

The assigned market prices for the cost-savings opportunities are displayed in Table 2 and explained in the following.

**Table 2:** Assigned monetary values to potential cost-saving opportunities

Categories	Potential cost savings	German market prices in €
Improved nurse-sensitive indicators	Fewer falls with injuries	Not available
	Fewer pressure ulcers category 2–4	At least €5,435 per pressure injury [41], no exact data available
Improved nursing personnel indicators	Lower nurse turnover rates	Not available
	Reduced use of agency nurses	On average 92% higher expenses for agency nurses (2022), individual hospital data* [42]
	Fewer sick days	Individual hospital data
Improved hospital performance	Reduction in the length of stay	Berlin: €1,070 per day (2023) [43]
	Revenues from additional nursing healthcare services	Individual hospital data
	Nurse driven cost saving initiatives	Individual hospital data

* When the monetization of cost savings from reduced nurse turnover takes agency nurses into account, cost savings from reduced use of agency nurses should be excluded to avoid double counting

#### **Nurse-sensitive indicators**

No economic studies were identified from the databases or grey literature that analyzed the costs of injurious falls or hospital-acquired pressure injuries in German or European hospitals. A cost approximation is given by seven medical aid supplier associations that published a position paper about the treatment of pressure injuries in 2016 [41]. They stated that for hospital acquired pressure injuries of categories 2 to 4, the minimum costs amount to 2.5 billion euros in Germany, with 460,000 people affected (ibid.). Calculated per patient, the minimum costs per pressure injury amount to €5,435.

#### **Nursing personnel figures**

Consistent with the absence of cost studies on nurse-sensitive indicators, no studies were identified on nurse turnover costs in German or European hospitals.

The Nursing Turnover Cost Calculation Methodology (NTCCM) by Jones can be used to identify all aspects necessary for calculating turnover costs [42]. It differentiates between pre- and posthire costs. Prehire costs consist of recruiting costs, vacancy costs, and hiring costs. Posthire costs include orientation, productivity, and termination costs.

A representative cross-sectional survey of 319 German hospitals in 2022 revealed that the average cost of agency nurses is 92% higher than the costs of employed nurses [43]. Individual hospital data should be used to indicate the exact cost-saving potential when reducing the number of agency nurses. Reductions in agency work due to ward closures should be disregarded when assessing the cost-saving potential. When using the NTCCM to calculate nurse turnover costs, agency costs should be excluded as they are already accounted for. 

Potential cost savings from a reduction in sickness days depend on how hospitals compensate for staff shortages. If sickness-related absences are covered by agency nurses, the associated cost savings are already reflected in the indicator ‘Reduced use of agency nurses’. Paid overtime generates additional personnel costs and should therefore be estimated using hospital-specific data. Productivity and efficiency losses resulting from understaffing are not taken into account.

#### **Hospital performance**

The statistical offices of the states in Germany publish an annual overview of hospital costs, which can be used to assign a value to the reduction in length of stay. Based on data from 2024, the cost per occupancy day was calculated at €1,140 for Berlin hospitals by the statistical office of Berlin-Brandenburg [44]. Hospital costs, consisting of personnel costs, material costs, interest, and taxes, as well as costs of training institutions were included and selected deductions, such as costs for scientific research and teaching, were subtracted (ibid.). Berlin was used as an illustrative example, and costs may vary across federal states depending on regional cost structures and hospital characteristics.

Potential revenues from the extension of nursing services as well as implemented nursing projects leading to possible cost savings are hospital-specific. Hence, no market prices can be assigned.

### Additional parameters required

The existing economic studies focusing on the U.S. or Saudi Arabia did not conduct a cost-benefit analysis [22–25] but focused on presenting business cases. For conducting and reporting a cost-benefit analysis, CHEERS should be followed [26].

First, the Magnet^®^ journey of hospitals spans several years. Therefore, it is necessary to adjust all costs and cost savings to a single base year, which should be the year in which Magnet^®^ designation is achieved. Future costs and cost savings should be discounted at an annual rate of 3%, in line with common health economic practice [45].

Second, to differentiate the effects of implementing Magnet^®^ from general healthcare sector trends, a comparison group is needed, such as hospitals not pursuing Magnet^®^ designation. Effects should be compared to identify those that can be included in the cost-benefit analysis. In Germany, public data on outcomes such as fall rates or staff turnover are not available; therefore, data collection by both Magnet^®^-aspiring/designated hospitals and comparable non-Magnet^®^ hospitals is required.

## Discussion

Relevant parameters for conducting a cost-benefit analysis of obtaining Magnet^®^ designation were identified and contextualized for German hospitals. Potential costs associated with pursuing Magnet^®^ include (i) fees of the ANCC, (ii) fees for benchmarking, (iii) expenses for professional development and staff and (iv) supporting resources, data management and innovations. Cost-saving opportunities associated with pursuing Magnet^®^ may arise from improvements in (i) nurse-sensitive indicators, (ii) nursing personnel indicators and (iii) hospital performance and were assigned monetary values, if available. A 3% discount rate for adjusting costs and cost savings was identified, and the lack of existing comparable data was noted.

### **Selection of potential costs and cost savings**

Conducting a literature review and using interviews with six representatives of five German hospitals ensured the identification of potential costs and benefits associated with pursuing Magnet^®^ designation relevant within the German healthcare system.

The cost selection in this study differs from previous research on the economic impact of Magnet^®^. This affects the inclusion of academization costs of nurses. A cross-sectional survey of nurses from 12 European countries published in 2012 already showed that none of the surveyed nurses in Germany held a bachelor’s degree, whereas in six other European countries, more than half of the practicing nurses had obtained the academic degree [46]. More recent evidence indicates that the academization rate of nursing in Germany has increased; however, it remains comparatively low. A study conducted in 2021 on the academization rate of nursing students in Germany found that only 1.75% were enrolled in bachelor programs [47]. In comparison, results from a randomized survey of nurses in the U.S. showed that over 70% held a bachelor’s degree or higher in 2022 [48]. Consequently, hospitals in Germany provide financial support for nurses to obtain higher qualifications in order to meet the Magnet^®^ requirements, whereas in other countries these costs are not typically associated with the implementation of the Magnet^®^ model. Nurses in leadership positions play a critical role in the implementation of the Magnet^®^ model [12]. To effectively fulfill this role, continuous professional development is required, as was pointed out by a qualitative study examining the character traits required of leaders in the context of Magnet^®^ implementation [49]. As indicated by interviewees, this may include coaching regarding the implementation of transformational leadership. Additionally, the inclusion of cost components related to supporting resources, data management, and innovations stands out from previous research. Implementation costs for data collection, extraction, and organization must be considered, given that in Germany data collection on nurse-sensitive indicators is legally required only for hospital-acquired pressure injuries [50]. In 2016, findings from a cross-sectional survey of 55 German hospitals with more than 500 beds indicated that nursing research was not integrated into over half of these hospitals [51]. To meet the Magnet^®^ requirements related to the implementation of evidence-based nursing practice and the advancement of nursing science [12], a prerequisite is access to the current developments in the field, facilitated, for example, through nursing-specific literature.

It is important to underscore the challenge of distinguishing general developments within the healthcare sector from expenditures specifically associated with Magnet^®^. For instance, the German Science and Humanities Council recommends an academization rate of 10–20% for nurses [52]. Interviewees also noted the possibility that their hospitals would have supported the academization of nurses without aiming for Magnet^®^ designation. Furthermore, the quality management policy set by the Federal Joint Committee requires German hospitals to regularly conduct surveys of patients and employees, though external administration is not mandatory [53]. A 2018 representative survey of 319 German hospitals found that approximately 87% conduct employee surveys to some extent [54], though average costs are not available. Therefore, only additional costs arising from Magnet^®^ implementation should be considered. For instance, if hospitals had already conducted external surveys for nurses or patients, participated in benchmarking for nurse-sensitive indicators, or covered nurses’ professional development costs exceeding the legal requirements, recommended by the German Hospital Federation [55], those costs would need to be deducted.

Magnet^®^ requires hospitals to provide data on four nurse-sensitive clinical indicators for inpatient units [35]. Two of the previous business cases, analyzing the impact of Magnet^®^, included four to five nurse-sensitive indicators [24, 25] and two focused on two nurse-sensitive indicators when assessing cost-saving potential [22, 23]. The expert standards for pressure injury and fall prevention, which German hospitals are required to implement, mandate the collection of data on hospital-acquired pressure injuries and falls [56, 57]. Also, the incidence of hospital-acquired pressure injuries is included as an indicator in the statutory quality assurance [50]. A qualitative analysis on the use of nurse-sensitive indicators in German hospitals showed limited availability of nurse-sensitive data [58]. Most interviewees reported collecting data on hospital-acquired pressure ulcers and falls (ibid.). Against this backdrop, this study focused on these two nurse-sensitive indicators due to data availability instead of the four required for data collection by Magnet^®^ [35], potentially reducing the possible benefits. When assessing nurse-sensitive clinical indicators, it is important to recognize that they capture only one part of the multidimensional concept of quality of care [59]. Hence, reductions in such outcome indicators should be evaluated not only in terms of potential cost savings, but also within the broader context of quality of care, including considerations of patient-centredness and ethical aspects of nursing care.

The average length of hospital stay for acute care patients in Germany ranked within the top third in international comparisons in 2021 [60]. Interviewees saw potential for a reduction in the length of stay when implementing Magnet^®^ due to improved patient care and organizational processes. Therefore, this aspect was included as a potential cost-saving indicator for German hospitals. However, it is equally important to critically consider the general development of a decrease in the length of stay in all German hospitals [61].

There are more differences in the selection of included costs and cost savings between the present study and already existing studies analyzing the economic benefit of obtaining Magnet^®^ designation. Some studies considered lower readmission rates or reduced needlestick injuries as cost-saving factors [23, 25]. Additionally, costs included in some analyses covered the optional fee-based document submission platform and fees for additional document submissions by the ANCC [24]. Since these expenses and cost savings were not identified as relevant by the interviewees involved in identifying relevant parameters for a cost-benefit analysis for German hospitals, these aspects were not considered.

### **Costs and cost-saving values**

The cost values can be collected directly from the hospitals, with the ANCC's costs being accessible on their website. The costs can be influenced by hospitals’ participation in Magnet4Europe. Interviewees stated that all participating hospitals in Magnet4Europe received Magnet^®^ materials free of charge. Additionally, the interviewees reported added value from their twinning partners - Magnet^®^-designated hospitals from the U.S. - that supported their hospital’s Magnet^®^ journey by sharing knowledge, best practices and experiences [19]. They also had free access to consulting support, for instance by Tipton Health, on the implementation of the Magnet^®^ principles [62].

The economic assessment of hospital-acquired pressure injuries and falls remains limited in research, especially with data from German hospitals. The provided costs of hospital-acquired pressure injuries, published by seven medical aid supplier associations, lack information on methodology and data sources, making them difficult to use [41]. Also, these costs refer to minimum expenditures, which do not allow for an exact cost determination of hospital-acquired pressure injuries. In international literature, a broad range of costs was determined for hospital-acquired pressure injuries. A pooled meta-analysis of economic studies on nurse-sensitive indicators was conducted by the Agency for Healthcare Research and Quality (AHRQ) [63]. Based on four economic studies on hospital-acquired pressure injuries, the AHRQ calculated costs of $14,506 per hospital-acquired pressure ulcer in 2015 (ibid.). A Markov simulation estimated an average cost of $10,708 in a 2016 economic analysis assessing the national burden of hospital-acquired pressure injuries in the U.S. This study highlighted the varying treatment costs depending on the severity of hospital-acquired pressure injuries, with category 3 and 4 incurring the highest expenses [64]. In the Czech Republic, a cost-of-illness study found the median cost of treating a pressure injury, including pharmacotherapy, amounting to €929 [65]. The study identified cost variations based on the category of pressure injuries, the patients’ age and the department providing care (ibid.). Based on a pooled meta-analysis of three studies, the AHRQ calculated costs of $6,694 for one fall in hospitals in 2015 [63]. A matched case-control study analyzing the costs of inpatient falls using U.S. data from 2013 to 2019 found that the average treatment cost of an inpatient fall without injury was $35,365, while the cost of an injurious fall averaged $36,776. These results indicate hardly any difference between the costs of injurious and non-injurious falls [66]. Existing economic studies that examine the costs and savings associated with Magnet^®^ designation are based on economic studies from the U.S [22–25]. However, due to structural differences between healthcare systems, these values cannot be transferred to the German healthcare sector. For instance, the American health care expenditures are higher than in Germany due to higher prices for healthcare services [67]. American health expenditures per capita amounted to $12,434.43, while expenditures in Germany amounted to $6,182.34 per capita in 2022 [68]. The different cost values of pressure injuries and injurious falls highlight the need for a comparative analysis of economic studies on falls and hospital-acquired pressure injuries and data generation for the German and European healthcare context.

Similar to the economic analyses of nurse-sensitive indicators, economic analyses of nursing turnover rates reveal high variability, highlighting the need for more research within the European, especially German, healthcare system. Literature reviews of calculated nursing turnover costs show discrepancies in the definition of turnover, data collection methods, and analytical approaches, leading to a wide range of cost estimates in different time periods from $10,098 to $88,000 per turnover [69–71].

Potential cost savings associated with reduced sickness days depend on hospital-specific replacement strategies. Hospitals may compensate for staff absences through agency nurses, paid overtime, internal staff redeployment, operating with reduced staffing levels, or temporary bed closures. Consequently, the potential financial impact of reduced sickness days varies substantially across settings and is best estimated using hospital-specific data. Nevertheless, reduced sickness absence may also generate productivity gains that are not directly reflected in hospital budgets. The Federal Institute for Occupational Safety and Health calculated production loss costs due to sickness with data from 2023. The underlying data for the calculation are based on the national accounts (Federal Statistical Office) and incapacity-to-work data from members of the statutory health insurance. The production loss costs for the public and other service providers, education, and health sector amount to €122 per day, based on an average gross salary of €52,300 [72]. While these estimates represent societal productivity losses rather than direct hospital costs, they help contextualize the potential economic impact of changes in sickness days. Future research should examine the economic impact of reduced sickness days from the hospital perspective, including hospital-specific replacement costs and potential productivity effects associated with staffing shortages or changes.

Furthermore, costs per occupancy day may vary across German federal states. While costs per occupancy day were calculated at €1,140 for Berlin in 2024 [44], they were calculated at €1,018 in 2024 for Bavaria [73].

When assessing the potential cost savings, it is important to consider the DRG-based reimbursement system. Within the DRG system, hospitals receive a fixed payment per case (§ 17b Hospital Financing Act). Hence, reductions in resource use do not automatically result in financial savings. Reductions in complications such as falls with injuries or pressure injuries may lower treatment costs; however, the extent to which these effects result in financial benefits depends on whether they affect DRG assignment or are accompanied by increased case throughput. Similarly, reductions in length of stay may decrease average costs per patient but do not necessarily result in direct financial savings, as a substantial share of hospital costs remains fixed [43]. Nurse-led innovations and extensions of nursing services may improve efficiency and quality of care, but their financial impact under DRG reimbursement depends on whether they translate into increased throughput, avoided costly complications, or other reimbursement-relevant effects. In contrast, staffing-related outcomes, such as reduced turnover, fewer sick days, or lower use of agency staff, are more directly associated with tangible cost reductions. These considerations should be taken into account when interpreting potential cost-saving values.

It is equally important to acknowledge that the valuation of potential costs and cost savings is influenced by the chosen perspective. While the present study adopts a hospital perspective, several of the identified cost-saving opportunities, particularly those related to nurse-sensitive indicators, sickness days, and length of stay, may generate additional value from a payer, patient, or societal perspective, including improved quality of life and reduced productivity losses.

### **Additional parameters to conduct a cost-benefit analysis**

Further parameters that need to be considered when conducting a cost-benefit analysis include discounting costs and savings to account for different time periods and having a comparison group to distinguish effects of Magnet^®^ implementation from general healthcare system developments.

Currently, conducting a cost-benefit analysis in Germany is hindered by the lack of available data. No hospitals have yet achieved Magnet^®^ designation, and only a few have committed to pursuing it, with each at different stages of implementation. Once these databases are established, a full cost-benefit analysis will become feasible. When selecting hospitals for a cost-benefit analysis, it is necessary to ensure that all hospitals provide cost-saving data in the same units, for example, sick days or turnover per person rather than a percentage in order to assign monetary values.

### **Practical application of the framework in hospitals**

Although data availability currently remains limited, the identified parameters for a cost-benefit analysis can still be utilized by hospitals through a stepwise approach to estimate the economic impact of Magnet^®^ implementation.

- In a first step, hospitals should map the identified relevant parameters against existing hospital information systems to determine which data are already available and which additional data flows need to be established.
- In a second step, relevant parameters should be systematically collected for each year during the implementation of the Magnet^®^ model.
- In a third step, identified non-monetary parameters need to be monetized by assigning unit costs. Given the limited availability of German cost data, hospitals could use internal data to estimate, for example, the costs of pressure injuries. This can be done using approaches such as propensity score matching, comparing patients with and without pressure injuries to estimate incremental costs. Alternatively, structured expert input from clinical nurses within the hospital may be used to complement missing information. Where available, nursing research units should be involved to support parameter estimation.
- All cost and savings components should be reported in a consistent annual format and, where appropriate, discounted using an annual rate of 3%.

Once hospitals begin to systematically build and harmonize such datasets, the framework also allows for conducting a cost-benefit analysis across hospitals, including undertaking sensitivity analyses to account for remaining variability in data quality and hospitals’ organizational context. Existing hospital data warehouses or information systems could potentially support this process by providing an infrastructure for data collection, which should be further explored in future research to improve data availability, harmonization, and transparency.

Assessing the economic impact of Magnet^®^ implementation is of high relevance, particularly given increasing financial pressures in the hospital sector as well as persistent nursing workforce shortages [2, 21]. Beyond overall cost-benefit considerations, the feasibility of implementation is likely to depend on initial investment requirements and the time needed to reach a potential break-even point for hospitals. This underlines the importance for hospitals on the Magnet^®^ journey to identify relevant data and establish appropriate data collection processes as outlined above, thereby addressing this research gap and enabling more informed economic decision-making in the future.

### **Limitations**

The following limitations have to be taken into account. It is an exploratory study identifying relevant parameters for a cost-benefit analysis based on literature findings and their contextualizing for the German healthcare system through expert interviews. The sample size of six interviewees from five German hospitals is intended to inform the identification of parameters relevant for a cost-benefit analysis but is not representative of all German hospitals. Interviews were conducted with individuals from hospitals participating in the Magnet4Europe study. These hospitals benefited from additional implementation support, such as twinning partnerships and consulting services provided at no cost. This was critically reflected in the interviews and cross-checked with findings from the literature. Nevertheless, this context may still lead to an underestimation of implementation costs compared with hospitals without access to similar support structures. The causality of the effects of Magnet^®^ in the German context has not been examined. The parameters for a cost-benefit analysis were identified from the provider’s perspective, as hospitals are responsible for deciding whether to pursue Magnet^®^ designation. Consequently, other perspectives - such as those of society, patients, or healthcare payers - were not considered, although they could influence the result of a cost-benefit analysis by focusing on a different set of costs and cost savings. Moreover, the present study focused solely on the impact of implementing the Magnet^®^ model on nursing, and the associated costs and benefits have been selected accordingly. This approach aligns with the methodology of previous business cases focusing on the U.S. or international contexts [22–25]. However, it cannot be ruled out that improvements also occur in other professional groups, such as in the retention or recruitment of doctors, possibly due to an improved interprofessional collaboration between these two occupational groups. This could lead to an underestimation of the cost-savings potential. Equity considerations, dealing with uncertainties, and exploring different investment criteria were not addressed in this theoretical study.

## Conclusion

This exploratory study identified potential expenses and cost-saving opportunities for German hospitals, providing relevant insights for hospital decision-makers and researchers when assessing the economic impact of Magnet^®^ designation. Monetary values were assigned to potential cost savings, highlighting implementation-relevant research gaps in the health economic assessment of nurse-sensitive indicators and turnover costs for Germany. Currently, conducting a cost-benefit analysis for German hospitals is impeded by the lack of available data. Harmonizing data collection processes is an important prerequisite for enhancing data comparability and should therefore be focused on in future research.

## Supplementary Information

Below is the link to the electronic supplementary material.

Supplementary Material 1

Supplementary Material 2

## Data Availability

The interview data that support the findings of this study are not publicly available due to the sensitivity of the qualitative interview material and the confidentiality assurances given to interviewees. Data may be available from the corresponding author upon reasonable request. All remaining data supporting the study’s results are embedded in the manuscript.

## Abbreviations

AHRQ: Agency for healthcare research and quality
ANCC: American nurses credentialing center
CHEERS: Consolidated health economic evaluation reporting standards
NTCCM: Nursing Turnover Cost Calculation Methodology

## References

[1] Sachverständigenrat zur Begutachtung der Entwicklung im Gesundheitswesen und in der Pflege. Fachkräfte im Gesundheitswesen: Nachhaltiger Einsatz einer knappen ressource. Gutachten 2024. Bonn (DE): Sachverständigenrat zur Begutachtung der Entwicklung im Gesundheitswesen und in der Pflege; 2024. 10.4126/FRL01-006473488.

[2] World Health Organization. State of the world's nursing 2020: investing in education, jobs and leadership [Internet]. Geneva: World Health Organization; 2020 [cited 2024 Dec 19]. Available from: https://iris.who.int/handle/10665/331677.

[3] Scheffler RM, Arnold DR. Projecting shortages and surpluses of doctors and nurses in the OECD: what looms ahead. Health Econ Policy Law. 2019;14(2):274–90. 10.1017/S174413311700055X. 29357954 10.1017/S174413311700055X

[4] Auffenberg J, Becka D, Evans M, Kokott N, Schleicher S, Braun E. „Ich pflege wieder, wenn …“ Eine Potenzialanalyse zur Berufsrückkehr und Arbeitszeitaufstockung von Pflegefachkräften [Internet]. Bremen (DE): Arbeitnehmerkammer Bremen; 2022 [cited 2024 Dec 19]. Available from: https://www.arbeitnehmerkammer.de/fileadmin/user_upload/Downloads/Politik/Rente_Gesundheit_Pflege/Bundesweite_Studie_Ich_pflege_wieder_wenn_Langfassung.pdf.

[5] Leiber S. Die Ankunft der Babyboomer: Was tun mit der „24-Stunden-Pflege“? In: Schwinger A, Kuhlmey A, Greß S, Klauber J, Jacobs K, Behrendt S, editors. Pflege-Report 2024 [Internet]. Berlin, Heidelberg: Springer Berlin Heidelberg; 2024 [cited 2024 Dec 21]. p. 87–98. Available from: 10.1007/978-3-662-70189-8_6.

[6] Sermeus W. Nurses' impact on quality of care: lessons from RN4CAST. Obz Zdr Nega. 2015;49(4). 10.14528/snr.2015.49.4.78.

[7] Copanitsanou P, Fotos N, Brokalaki H. Effects of work environment on patient and nurse outcomes. Br J Nurs. 2017;26(3):172–6. 10.12968/bjon.2017.26.3.172. 28185485 10.12968/bjon.2017.26.3.172

[8] Aiken LH, Lasater KB, Sloane DM, Pogue CA, Fitzpatrick Rosenbaum KE, Muir KJ, et al. Physician and Nurse Well-Being and Preferred Interventions to Address Burnout in Hospital Practice: Factors Associated With Turnover, Outcomes, and Patient Safety. JAMA Health Forum. 2023;4(7):e231809. 10.1001/jamahealthforum.2023.1809. 37418269 10.1001/jamahealthforum.2023.1809 PMC10329209

[9] Perry SJ, Richter JP, Beauvais B. The Effects of Nursing Satisfaction and Turnover Cognitions on Patient Attitudes and Outcomes: A Three-Level Multisource Study. Health Serv Res. 2018;53(6):4943–69. 10.1111/1475-6773.12997. 29957888 10.1111/1475-6773.12997 PMC6232403

[10] IEGUS, contec, WifOR. Arbeitsplatzsituation in der Akut- und Langzeitpflege und Ermittlung sowie modellhafte Implementierung von Indikatoren für gute Arbeitsbedingungen in der Langzeitpflege – Los 1: analyse, Befragungen und Maßnahmenempfehlungen zum Pflegearbeitsplatz der Zukunft [Internet]. 2022 [cited 2025 May 5]. Available from: https://www.bundesgesundheitsministerium.de/fileadmin/Dateien/3_Downloads/K/Konzertierte_Aktion_Pflege/Abschlussbericht_Studie_Arbeitsplatzsituation_in_der_Akut-_und_Langzeitpflege_Los-1_barrierefrei.pdf.

[11] American nurses credentialing center (ANCC). About Magnet [Internet]. Silver spring (MD): American nurses credentialing center; 2025 [cited 2025 May 5]. Available from: https://www.nursingworld.org/organizational-programs/magnet/about-magnet/.

[12] American nurses credentialing center (ANCC). Magnet model: creating a magnet culture [Internet]. Silver spring (MD): American nurses credentialing center; 2025 [cited 2025 May 5]. Available from: https://www.nursingworld.org/organizational-programs/magnet/magnet-model/.

[13] American nurses credentialing center (ANCC). Find a magnet organization [Internet]. Silver spring (MD): American nurses credentialing center; 2025 [cited 2025 May 5]. Available from: https://www.nursingworld.org/organizational-programs/magnet/find-a-magnet-organization/.

[14] Aiken LH, Havens DS, Sloane DM. The Magnet Nursing Services Recognition Program: A Comparison of Two Groups of Magnet Hospitals. JONA J Nurs Adm. 2009;39(7/8):S5–14. 10.1097/NNA.0b013e3181aeb469. 10.1097/NNA.0b013e3181aeb469 PMC4452009 19641439

[15] Hamadi HY, Martinez D, Palenzuela J, Spaulding AC. Magnet Hospitals and 30-Day Readmission and Mortality Rates for Medicare Beneficiaries. Med Care. 2021;59(1):6–12. 10.1097/MLR.0000000000001427. 32925454 10.1097/MLR.0000000000001427

[16] Kelly LA, McHugh MD, Aiken LH. Nurse Outcomes in Magnet^®^ and Non-Magnet Hospitals. JONA J Nurs Adm. 2012;42(10):S44–9. 10.1097/01.NNA.0000420394.18284.4f. 10.1097/01.NNA.0000420394.18284.4f PMC5606241 22976894

[17] Rodríguez-García MC, Márquez-Hernández VV, Belmonte-García T, Gutiérrez-Puertas L, Granados-Gámez G. Original Research: How Magnet Hospital Status Affects Nurses, Patients, and Organizations: A Systematic Review. AJN Am J Nurs. 2020;120(7):28–38. 10.1097/01.NAJ.0000681648.48249.16. 32541337 10.1097/01.NAJ.0000681648.48249.16

[18] Magnet4Europe. The project – at a glance [Internet]. 2024 [cited 2025 May 5]. Available from: https://www.magnet4europe.eu/at-a-glance.html.

[19] Sermeus W, Aiken LH, Ball J, Bridges J, Bruyneel L, Busse R, et al. A workplace organisational intervention to improve hospital nurses’ and physicians’ mental health: study protocol for the Magnet4Europe wait list cluster randomised controlled trial. BMJ Open. 2022;12(7):e059159. 10.1136/bmjopen-2021-059159. 35902190 10.1136/bmjopen-2021-059159 PMC9341186

[20] Accenture. Europe’s hospitals: an industry operating in the red [Internet]. 2014 [cited 2024 Dec 21]. Available from: http://eahm.eu.org/wp-content/uploads/2016/04/Accenture-European-Hospitals-An-Industry-Operating-In-The-Red.pdf.

[21] German hospital institute. KRANKENHAUS BAROMETER Umfrage 2023 [Internet]. 2023. Available from: https://www.dkgev.de/fileadmin/default/Mediapool/1_DKG/1.7_Presse/1.7.1_Pressemitteilungen/2023/2023-12-27_PM_Anlage_DKI-Krankenhaus-Barometer_2023.pdf.

[22] Drenkard K. The Business Case for Magnet^®^. JONA J Nurs Adm. 2010;40(6):263–71. 10.1097/NNA.0b013e3181df0fd6. 10.1097/NNA.0b013e3181df0fd6 20502195

[23] Higdon K, Clickner D, Gray F, Woody G, Shirey M. Business Case for Magnet^®^ in a Small Hospital. JONA J Nurs Adm. 2013;43(2):113–8. 10.1097/NNA.0b013e31827f2208. 10.1097/NNA.0b013e31827f2208 23343728

[24] February T, Holmes S. The Business Case for Magnet^®^ in an International Hospital. JONA J Nurs Adm. 2020;50(10):533–8. 10.1097/NNA.0000000000000930. 10.1097/NNA.0000000000000930 32925664

[25] Drenkard K. The Business Case for Magnet^®^ Designation: Using Data to Support Strategy. JONA J Nurs Adm. 2022;52(9):452–61. 10.1097/NNA.0000000000001182. 10.1097/NNA.0000000000001182 PMC9415212 35973435

[26] Husereau D, Drummond M, Augustovski F, Briggs A, Carswell C, Caulley L, et al. Consolidated Health Economic Evaluation Reporting Standards 2022 (CHEERS 2022) statement: updated reporting guidance for health economic evaluations. BJOG Int J Obstet Gynaecol. 2022;129(3):336–44. 10.1111/1471-0528.17012. 10.1111/1471-0528.17012 35014160

[27] Grant MJ, Booth A. A typology of reviews: an analysis of 14 review types and associated methodologies. Health Inf Libr J. 2009;26(2):91–108. 10.1111/j.1471-1842.2009.00848.x. 10.1111/j.1471-1842.2009.00848.x 19490148

[28] Gurisch C, Kleine J, Maier CB. International models of accreditation and certification for hospitals with a focus on nursing: a scoping review. BMC Health Serv Res. 2024;24(1):1385. 10.1186/s12913-024-11759-6. 39533263 10.1186/s12913-024-11759-6 PMC11559163

[29] Booth A. Clear and present questions: formulating questions for evidence based practice. In: Cleyle S, editor. Libr Hi Tech. 2006;24(3):355–368. 10.1108/07378830610692127.

[30] Malterud K, Siersma VD, Guassora AD. Sample Size in Qualitative Interview Studies: Guided by Information Power. Qual Health Res. 2016;26(13):1753–60. 10.1177/1049732315617444. 26613970 10.1177/1049732315617444

[31] Mishan EJ, Quah E. Cost-benefit analysis. Sixth edition. Abingdon, Oxon: Routledge; 2021.

[32] American nurses credentialing center (ANCC). Magnet application and appraisal fees (starting January 1, 2022) [Internet]. Silver spring (MD): American nurses credentialing center; 2021 [cited 2025 May 5]. Available from: https://www.nursingworld.org/organizational-programs/magnet/apply/magnet-fees/2022/.

[33] American nurses credentialing center (ANCC). Pre-intent program [Internet]. Silver spring (MD): American nurses credentialing center; 2025 [cited 2025 May 5]. Available from: https://www.nursingworld.org/organizational-programs/magnet/program-tools/pre-intent-program/.

[34] ANCC. 2023 Magnet^®^ Application Manual [Internet]. 2025. Available from: https://www.nursingworld.org/nurses-books/2023-magnet-application-manual2/?srsltid=AfmBOor2xqtgGE2T-4MBCqtVvBH3NPo0c9IlLqaR1cZhSu2OSVKrVOCM

[35] American nurses credentialing center (ANCC). 2023 Magnet® application manual [Effective: August 1, 2024]: international interpretation of selected sources of evidence [Internet]. Silver spring (MD): American nurses credentialing center; 2024 [cited 2025 May 5]. Available from: https://www.nursingworld.org/globalassets/organizational-programs/magnet/international/v4-2023-magnet-application-manual---intl-interpretation-of-select-soes--aug-2024.pdf.

[36] American nurses credentialing center (ANCC). Accepted database vendors [Internet]. Silver spring (MD): American nurses credentialing center; 2025 [cited 2025 Dec 16]. Available from: https://www.nursingworld.org/organizational-programs/magnet/program-tools/accepted-certifications/accepted-database-vendors/.

[37] American nurses credentialing center (ANCC). ANCC national magnet conference [Internet]. Silver spring (MD): American nurses credentialing center; 2025 [cited 2025 May 5]. Available from: https://magnetpathwaycon.nursingworld.org/.

[38] Lake ET, Shang J, Klaus S, Dunton NE. Patient falls: association with hospital magnet status and nursing unit staffing. Res Nurs Health. 2010;33(5):413–25. 10.1002/nur.20399. 20824686 10.1002/nur.20399 PMC2974438

[39] Ma C, Park SH. Hospital Magnet Status, Unit Work Environment, and Pressure Ulcers. J Nurs Scholarsh. 2015;47(6):565–73. 10.1111/jnu.12173. 26474091 10.1111/jnu.12173

[40] Goode CJ, Blegen MA, Park SH, Vaughn T, Spetz J. Comparison of Patient Outcomes in Magnet^®^ and Non-Magnet Hospitals. JONA J Nurs Adm. 2011;41(12):517–23. 10.1097/NNA.0b013e3182378b7c. 10.1097/NNA.0b013e3182378b7c 22094616

[41] BVMed, Fachvereinigung Medizinprodukte (f.m.p.), Reha-Service-Ring (RSR), rehaVital Gesundheitsservice GmbH, EGROH eG, Orthopädie.Technik, et al. Dramatischer Kostenanstieg durch Fehlentwicklungen in der Dekubitusversorgung [Internet]. Berlin (DE): Bundesverband Medizintechnologie (BVMed); 2016 [cited 2025 Dec 16]. Available from: https://www.ot-nord.de/v1/uploads/Aktuelles/170113_positionspapier-antidekubitusversorgung-dezember-2016.pdf.

[42] Jones CB. The Costs of Nurse Turnover, Part 2: Application of the Nursing Turnover Cost Calculation Methodology. JONA J Nurs Adm. 2005;35(1):41–9. 10.1097/00005110-200501000-00014. 10.1097/00005110-200501000-00014 15647669

[43] Deutsches Krankenhausinstitut (DKI). DKI Krankenhaus-Pool [Internet]. Düsseldorf (DE): Deutsches Krankenhausinstitut e.V.; 2022 [cited 2025 Dec 16]. Available from: https://www.dkgev.de/fileadmin/default/Mediapool/1_DKG/1.7_Presse/1.7.1_Pressemitteilungen/2023/Anlage_DKI-Umfrage_Krankenhaus-Pool_Leiharbeit_im_Krankenhaus.pdf.

[44] Amt für Statistik Berlin-Brandenburg. Krankenhäuser im Land Berlin 2024 [Internet]. Potsdam (DE): Amt für Statistik Berlin-Brandenburg; 2026 [cited 2025 Dec 16]. Available from: https://download.statistik-berlin-brandenburg.de/d55546713f576f5a/0379d21ffed5/SB_A04-04-00_2024j01_BE.pdf.

[45] Institut für Qualität und Wirtschaftlichkeit im Gesundheitswesen (IQWiG). Allgemeine Methoden (Entwurf für Version 8.0) [Internet]. Köln (DE): IQWiG; 2025 [cited 2025 Dec 16]. Available from: https://www.iqwig.de/methoden/allgemeine-methoden_entwurf-fuer-version-8-0.pdf.

[46] Aiken LH, Sloane DM, Bruyneel L, Van Den Heede K, Sermeus W. Nurses’ reports of working conditions and hospital quality of care in 12 countries in Europe. Int J Nurs Stud. 2013;50(2):143–53. 10.1016/j.ijnurstu.2012.11.009. 23254247 10.1016/j.ijnurstu.2012.11.009

[47] Meng M, Peters M, Dorin L. Erste Sondererhebung des BIBB-Pflegepanels: ein aktueller Überblick zu berufsqualifizierenden Pflegestudiengängen [Internet]. Version 1.0. Bonn (DE): Bundesinstitut für Berufsbildung (BIBB); 2022 [cited 2025 May 5]. Available from: https://res.bibb.de/vet-repository_780291.

[48] Smiley RA, Allgeyer RL, Shobo Y, Lyons KC, Letourneau R, Zhong E, et al. The 2022 National Nursing Workforce Survey. J Nurs Regul. 2023;14(1):S1–90. 10.1016/S2155-8256(23)00047-9.37012978

[49] Kleine J, Köppen J, Gurisch C, Maier CB. Transformational nurse leadership attributes in German hospitals pursuing organization-wide change via Magnet^®^ or Pathway^®^ principles: results from a qualitative study. BMC Health Serv Res. 2024;24(1):440. 10.1186/s12913-024-10862-y. 38589915 10.1186/s12913-024-10862-y PMC11003170

[50] Institut für Qualitätssicherung und Transparenz im Gesundheitswesen (IQTIG). Dekubitusprophylaxe (QS DEK) [Internet]. Berlin (DE): IQTIG; 2025 [cited 2025 May 5]. Available from: https://iqtig.org/qs-verfahren/qs-dek/.

[51] Horbach A, Berendonk C, von Borstel B, Fink-Heitz M, Imbery C, Knipfer E, et al. Wo steht die klinische Pflegeforschung? [Internet]. 2016 [cited 2025 Nov 13]. Available from: https://www.bibliomed-pflege.de/news/29550-wo-steht-die-klinische-pflegeforschung.

[52] German Science and Humanities Council. Recommendations on higher education qualifications for the healthcare system [Internet]. 2012. Available from: https://www.wissenschaftsrat.de/download/archiv/2411-12_Executive-Summary.pdf?__blob=publicationFile&v=6

[53] Gemeinsamer Bundesausschuss (G-BA). Richtlinie des Gemeinsamen Bundesausschusses über grundsätzliche Anforderungen an ein einrichtungsinternes Qualitätsmanagement für Vertragsärztinnen und Vertragsärzte, Vertragspsychotherapeutinnen und Vertragspsychotherapeuten, medizinische Versorgungszentren, Vertragszahnärztinnen und Vertragszahnärzte sowie zugelassene Krankenhäuser (Qualitätsmanagement-Richtlinie/QM-RL) [Internet]. Berlin (DE): Gemeinsamer Bundesausschuss; 2024 [cited 2025 Dec 16]. Available from: https://www.g-ba.de/downloads/62-492-3427/QM-RL_2024-01-18_iK-2024-04-20.pdf.

[54] Deutsches Krankenhausinstitut (DKI), BDO AG Wirtschaftsprüfungsgesellschaft. Das Krankenhaus als attraktiver Arbeitgeber [Internet]. Düsseldorf (DE): Deutsches Krankenhausinstitut e.V.; 2018 [cited 2025 Dec 16]. Available from: https://www.dki.de/fileadmin/forschungsberichte/Das_Krankenhaus_als_attraktiver_Arbeitgeben.pdf.

[55] Deutsche Krankenhausgesellschaft (DKG). DKG-Empfehlung zur pflegerischen Fachweiterbildung [Internet]. Berlin (DE): Deutsche Krankenhausgesellschaft e.V.; 2023 [cited 2025 Dec 16]. Available from: https://www.dkgev.de/fileadmin/default/Mediapool/2_Themen/2.5._Personal_und_Weiterbildung/2.5.11._Aus-_und_Weiterbildung_von_Pflegeberufen/Pflegerische_Weiterbildung/Downloads_ab_04.07.2023/DKG-Empfehlung_Pflegerische_Fachweiterbildungen.pdf.

[56] Büscher A, Blumenberg P, Krebs M, Stehling H, Stomberg D. Expertenstandard Dekubitusprophylaxe in der Pflege: 2. Aktualisierung 2017. Osnabrück (DE): Hochschule Osnabrück, Fakultät für Wirtschafts- und Sozialwissenschaften; 2021. 116 p. (Schriftenreihe des Deutschen Netzwerks für Qualitätsentwicklung in der Pflege).

[57] Büscher A, Blumenberg P, Krebs M, Niemann LM, Stehling H, Stomberg D. Expertenstandard Sturzprophylaxe in der Pflege: 2. Aktualisierung 2022. Osnabrück (DE): Hochschule Osnabrück, Fakultät für Wirtschafts- und Sozialwissenschaften; 2022. 116 p. (Schriftenreihe des Deutschen Netzwerks für Qualitätsentwicklung in der Pflege).

[58] Maier CB, Gurisch C, Köppen J, Kleine J, Aiken LH. Nurse-sensitive quality and benchmarking in hospitals striving for Magnet® or Pathway® designation: a qualitative study. J Adv Nurs. 2024;16245. 10.1111/jan.16245. 10.1111/jan.16245 PMC12371794 38803125

[59] McEvoy NL, Kalvas LB, Walsh K, Curley MAQ. The identification and characterization of nurse-sensitive outcomes in acute and critical care: A systematic review. Nurs Outlook. 2025;73(2):102379. 10.1016/j.outlook.2025.102379. 39999613 10.1016/j.outlook.2025.102379

[60] eurostat. Hospital discharges and length of stay statistics [Internet]. 2025. Available from: https://ec.europa.eu/eurostat/statistics-explained/index.php?title=Hospital_discharges_and_length_of_stay_statistics

[61] Statista. Average length of stay in hospitals in Germany from 1992 to 2023 [Internet]. Hamburg (DE): Statista GmbH; 2024 [cited 2025 Dec 16]. Available from: https://www.statista.com/statistics/578489/hospital-length-of-stay-germany/.

[62] Magnet4Europe, Tipton health. Applying magnet to your service line workshop [Internet]. 2021 [cited 2025 Dec 16]. Available from: https://www.magnet4europe.eu/blog-page/tipton-health-donates-50000-in-educational-support-to-magnet4europe.

[63] Agency for healthcare research and quality (AHRQ). Estimating the additional hospital inpatient cost and mortality associated with selected hospital-acquired conditions [Internet]. Rockville (MD): agency for healthcare research and quality; 2017 [cited 2025 Dec 16]. Available from: https://www.ahrq.gov/hai/pfp/haccost2017-results.html.

[64] Padula WV, Delarmente BA. The national cost of hospital-acquired pressure injuries in the United States. Int Wound J. 2019;16(3):634–40. 10.1111/iwj.13071. 30693644 10.1111/iwj.13071 PMC7948545

[65] Krupová L, Pokorná A, Krupa M, Benešová K. Comprehensive cost-of‐illness analysis of pressure ulcer treatment: A real‐world study at a Czech university hospital. Int Wound J. 2025;22(1):e70137. 10.1111/iwj.70137. 39800361 10.1111/iwj.70137 PMC11725365

[66] Dykes PC, Curtin-Bowen M, Lipsitz S, Franz C, Adelman J, Adkison L, et al. Cost of Inpatient Falls and Cost-Benefit Analysis of Implementation of an Evidence-Based Fall Prevention Program. JAMA Health Forum. 2023;4(1):e225125. 10.1001/jamahealthforum.2022.5125. 36662505 10.1001/jamahealthforum.2022.5125 PMC9860521

[67] Organisation for economic co-operation and development (OECD). Understanding differences in health expenditure between the United States and OECD countries [Internet]. Paris (FR): OECD publishing; 2022 [cited 2025 Dec 16]. Available from: https://www.oecd.org/content/dam/oecd/en/publications/reports/2022/09/understanding-differences-in-health-expenditure-between-the-united-states-and-oecd-countries_cafc404c/6f24c128-en.pdf.

[68] World Health Organization (WHO). Current health expenditure (CHE) per capita in US$ [Internet]. Geneva (CH): World Health Organization; 2025 [cited 2025 Dec 16]. Available from: https://www.who.int/data/gho/data/indicators/indicator-details/GHO/current-health-expenditure-(che)-per-capita-in-us-dollar.

[69] Bae S. Noneconomic and economic impacts of nurse turnover in hospitals: A systematic review. Int Nurs Rev. 2022;69(3):392–404. 10.1111/inr.12769. 35654041 10.1111/inr.12769 PMC9545246

[70] Li Y, Jones CB. A literature review of nursing turnover costs: *A Literature review of nursing turnover costs*. J Nurs Manag. 2013;21(3):405–18. 10.1111/j.1365-2834.2012.01411.x. 23406301 10.1111/j.1365-2834.2012.01411.x

[71] NSI Nursing Solutions, Inc. 2025 NSI national health care retention & RN staffing report [Internet]. East Petersburg (PA): NSI nursing solutions, Inc.; 2025 [cited 2025 Dec 16]. Available from: https://www.nsinursingsolutions.com/documents/library/nsi_national_health_care_retention_report.pdf.

[72] Federal institute for occupational safety and health (BAuA). Volkswirtschaftliche Kosten durch Arbeitsunfähigkeit 2023 [Internet]. Dortmund (DE): Bundesanstalt für Arbeitsschutz und Arbeitsmedizin; 2024 [cited 2025 Dec 16]. Available from: https://www.baua.de/DE/Themen/Monitoring-Evaluation/Zahlen-Daten-Fakten/Kosten-der-Arbeitsunfaehigkeit.html.

[73] Bayerisches Landesamt für Statistik. Krankenhausstatistik 2024: Grunddaten, Diagnosen und Kostennachweis [Internet]. Fürth (DE): Bayerisches Landesamt für Statistik; 2025 [cited 2025 Dec 16]. Available from: https://www.statistik.bayern.de/mam/produkte/veroffentlichungen/statistische_berichte/a4200c_202400.pdf.

