# Supplementary material for: The economics of implementing Magnet® designation: relevant parameters for a cost-benefit analysis in the German hospital context
Source: BMC Health Serv Res. 2026 Aug 12;26:1142. doi: 10.1186/s12913-026-15346-9 (PMC13479451; doi:10.1186/s12913-026-15346-9)
Supplement: Supplementary file 1 [file 12913_2026_15346_MOESM1_ESM.docx]

Appendix A: Semi-structured interview guide

Study title: The economics of implementing Magnet® designation: Relevant parameters for a cost-benefit analysis in the German hospital context

1. **Introduction**

- Greeting
- Introduction of the interviewer
- Aim of the interviews:
  - This interview focuses on your experiences and assessments of (potential) costs and cost-savings related to Magnet implementation.
  - The results will be compared with findings from the international literature.
- Background information:
  - Duration of the interview:
    - The interview will last between 45 and 60 minutes. You are invited to share everything that comes to your mind in response to the questions.
    - You may ask questions at any time.
    - Of course, there are no negative consequences if you choose to end the interview early.
  - Anonymity:
    - Your statements in this interview will be treated with complete confidentiality. The results will be summarized across all interviews, prepared for a scientific publication, and possibly presented at scientific conferences.
    - No conclusions can be drawn about your identity or your hospital.
  - Consent to audio recording:
    - This interview will be recorded. Do you agree to that?

**>>>>>> After agreement, start of audio recording of the interview <<<<<<<<<**

2. **Ice-Breaker**

- What is your hospital’s current status regarding the implementation of the Magnet concept?

3. **(Potential) cost-savings**

- What positive effects has the implementation of the Magnet concept had in your hospital?

*Probing*:

- *Patient-related outcomes (e.g., reduction of adverse events such as falls, pressure ulcers, infections)*
- *Nursing-related outcomes (e.g., reduced staff turnover)*
- *Hospital performance (e.g., improved performance indicators)*
- Can you estimate the financial magnitude of each of these effects?

*Probing*:

- *Can you provide concrete examples?*
- *Have you observed measurable changes?*
- *Financial impact of improvement of reduction of adverse events*
- *Financial impact of improvement of nursing-related outcomes*
- *Financial impact of improved performance indicators*
- *Data used internally*
- *Over what time period do these effects occur?*
- What additional benefits do you expect in the future?
- Can you estimate the financial magnitude of these benefits in the future?

4. **Costs**

- What costs have arisen from implementing the Magnet concept in your hospital?

*Probing*:

- *Designation-related costs (e.g., ANCC)*
- *benchmarking-related costs*
- *Organizational changes (e.g., additional staff needed)*
- Can you estimate the level of each of these costs?

*Probing*:

- *Designation-related costs (e.g., ANCC)*
- *benchmarking-related costs*
- *Organizational changes (e.g., additional staff needed)*
- *Data used internally*
- *Are these one-time or ongoing costs?*
- What additional costs do you expect in the future?
- Can you estimate the magnitude of these additional costs in the future?

5. **End**

- Are there any questions or comments you want to add?

**>>>>>>>>>>>>>>>>>>> Stop Audio recording >>>>>>>>>>>>>>>>>>>>>**

- Thank you very much for your time and your insights!
- Collection of sociodemographic data:
  - Job title
  - Work experience in years
  - Experience with Magnet implementation in years
  - Hospital’s status of pursuing Magnet designation
