# Supplementary material for: The economics of implementing Magnet® designation: relevant parameters for a cost-benefit analysis in the German hospital context
Source: BMC Health Serv Res. 2026 Aug 12;26:1142. doi: 10.1186/s12913-026-15346-9 (PMC13479451; doi:10.1186/s12913-026-15346-9)
Supplement: Supplementary file 2 [file 12913_2026_15346_MOESM2_ESM.docx]

Appendix B: Potential costs and cost-saving opportunities – evidence from literature, relevance in interviews, definition

| **Aspect** | **Evidence from literature** | | **Relevance in interviews (x=yes, (x)=partial, -=no)** | **Definition** |
| --- | --- | --- | --- | --- |
| **Costs** | | | | |
| **Fees by ANCC** |  |  |  |  |
| Pre-Intent Program Subscription | February & Holmes, 2020 | The Business Case for Magnet® in an International Hospital | (x) | Magnet® offers a voluntary pre-intent program; in case of participation, the costs should be included. |
| Magnet® material | Higdon et al., 2013 | Business case for Magnet® in a small hospital | (x)  (already covered through participation in Magnet4Europe) | Expenses for the purchase of Magnet® materials, such as the Magnet® manual or Magnet® principles related manuals. |
| Application fees | Drenkard, 2010 | The Business Case for Magnet® | x | Fee for applying for Magnet® designation. |
|  | Drenkard, 2022 | The Business Case for Magnet® Designation: Using Data to Support Strategy |  |  |
|  | February & Holmes, 2020 | The Business Case for Magnet® in an International Hospital |  |  |
|  | Higdon et al., 2013 | Business case for Magnet® in a small hospital |  |  |
| Appraiser fees | Drenkard, 2010 | The Business Case for Magnet® | x | Costs for the appraisers. |
|  | Drenkard, 2022 | The Business Case for Magnet® Designation: Using Data to Support Strategy |  |  |
|  | February & Holmes, 2020 | The Business Case for Magnet® in an International Hospital |  |  |
|  | Higdon et al., 2013 | Business case for Magnet® in a small hospital |  |  |
| Site-visit costs | Drenkard, 2010 | The Business Case for Magnet® | x | Costs for the appraisers for travelling to hospital to review fulfillment of Magnet® requirements. |
|  | Drenkard, 2022 | The Business Case for Magnet® Designation: Using Data to Support Strategy |  |  |
|  | February & Holmes, 2020 | The Business Case for Magnet® in an International Hospital |  |  |
|  | Higdon et al., 2013 | Business case for Magnet® in a small hospital |  |  |
| Document submission | Drenkard, 2022 | The Business Case for Magnet® Designation: Using Data to Support Strategy | - | Expenses for the submission platform or for the subsequent submission of additional documents |
|  | February & Holmes, 2020 | The Business Case for Magnet® in an International Hospital |  |  |
| **Fees for benchmarking** |  |  |  |  |
| Subscription to benchmarking of nurse-sensitive indicators | February & Holmes, 2020 | The Business Case for Magnet® in an International Hospital | x | Costs for participation in external benchmarking of nurse-sensitive indicators, in accordance with Magnet® designation requirements. If there was prior participation in regular external benchmarking of patient safety indicators, which was then replaced, only the additional costs should be included. |
|  | Higdon et al., 2013 | Business case for Magnet® in a small hospital |  |  |
| Subscription to benchmarking of patient satisfaction | February & Holmes, 2020 | The Business Case for Magnet® in an International Hospital | x | Costs for the survey of patients, in accordance with Magnet® designation requirements. If a regular patient survey was already conducted beforehand and is now replaced, only the additional costs should be included. |
| Subscription to benchmarking of nurse satisfaction | February & Holmes, 2020 | The Business Case for Magnet® in an International Hospital | x | Costs for the survey of nurses, in accordance with Magnet® designation requirements. If a regular survey of nurses was already conducted beforehand and is now replaced, only the additional costs should be included. |
| **Hospitals costs for professional development and staff** |  |  |  |  |
| Professional development opportunities for nurses | February & Holmes, 2020 | The Business Case for Magnet® in an International Hospital | x | Expenses for the professional development of nurses according to the Magnet® requirements.  If legal requirements for nurses' development are applied as part of fulfilling the Magnet® requirements, these expenses should be excluded. |
| Professional development opportunities for leadership (transformational leadership) | - |  | x | Expenses for the training of leadership, for instance in transformational leadership. |
| Attendance of Magnet® conferences | Higdon et al., 2013 | Business case for Magnet® in a small hospital | x | Travel and participation expenses for Magnet® conferences. |
| Academization of nurses | - |  | x | Expenses for the academic advancement of nursing staff in the form of covering study costs. |
| Staff costs | February & Holmes, 2020 | The Business Case for Magnet® in an International Hospital | (x)  (Some hospitals reported no additional staffing, as Magnet® was implemented within existing structures) | Personnel costs for the coordination and implementation of Magnet® |
|  | Higdon et al., 2013 | Business case for Magnet® in a small hospital |  |  |
| **Costs for supporting resources, data management, and innovations** |  |  |  |  |
| Implementation costs for data collection, data extraction, data organization | - |  | x | Expenses for software/licenses required for the necessary data collection or implementation of the Magnet® principles. |
| Access to nursing-specific literature | - |  | x | Expenses for access to nursing-specific literature. In case of access to the literature independently of the Magnet® journey, these costs should be excluded or additional costs only included. |
| Costs for the implementation of innovations | February & Holmes, 2020 | The Business Case for Magnet® in an International Hospital | x | Expenses for the implementation of nurse-led innovations. |
| Involvement of external consultants | Drenkard, 2010 | The Business Case for Magnet® | (x)  (not every hospital plans involvement of consultants) | Costs for external consultants who assist, e.g., with the gap analysis or document preparation/review. |
|  | Drenkard, 2022 | The Business Case for Magnet® Designation: Using Data to Support Strategy |  |  |
|  | February & Holmes, 2020 | The Business Case for Magnet® in an International Hospital |  |  |
|  | Higdon et al., 2013 | Business case for Magnet® in a small hospital |  |  |
| **Cost savings** | | | | |
| **Nurse-sensitive indicators** |  |  |  |  |
| Fewer pressure ulcers category 2 to 4 | Drenkard, 2010 | The Business Case for Magnet® | (x) | Revenues from the reduction of hospital-acquired pressure injuries category 2 to 4. |
|  | Drenkard, 2022 | The Business Case for Magnet® Designation: Using Data to Support Strategy |  |  |
|  | February & Holmes, 2020 | The Business Case for Magnet® in an International Hospital |  |  |
|  | Goode et al., 2011 | Comparison of patient outcomes in Magnet® and non-Magnet hospitals |  |  |
|  | Higdon et al., 2013 | Business case for Magnet® in a small hospital |  |  |
|  | Ma & Park, 2015 | Hospital Magnet Status, Unit Work Environment, and Pressure Ulcers |  |  |
|  | Petit Dit Dariel & Regnaux, 2015 | Do Magnet®-accredited hospitals show improvements in nurse and patient outcomes compared to non-Magnet hospitals: a systematic review |  |  |
|  | Rodríguez-García et al., 2020 | How Magnet Hospital Status Affects Nurses, Patients, and Organizations: A Systematic Review |  |  |
| Fewer falls with injuries | Connor et al., 2023 | The Association Between Magnet ® Recognition and Patient Outcomes : A Scoping Review | (x) | Revenues from the reduction of falls with injury. |
|  | Drenkard, 2010 | The Business Case for Magnet® |  |  |
|  | Drenkard, 2022 | The Business Case for Magnet® Designation: Using Data to Support Strategy |  |  |
|  | February & Holmes, 2020 | The Business Case for Magnet® in an International Hospital |  |  |
|  | Higdon et al., 2013 | Business case for Magnet® in a small hospital |  |  |
|  | Lake et al., 2010 | Patient falls: Association with hospital Magnet status and nursing unit staffing |  |  |
|  | Petit Dit Dariel & Regnaux, 2015 | Do Magnet®-accredited hospitals show improvements in nurse and patient outcomes compared to non-Magnet hospitals: a systematic review |  |  |
|  | Rodríguez-García et al., 2020 | How Magnet Hospital Status Affects Nurses, Patients, and Organizations: A Systematic Review |  |  |
| Fewer catheter-associated urinary tract infections (CAUTI) | Drenkard, 2022 | The Business Case for Magnet® Designation: Using Data to Support Strategy | - | Revenues from the reduction of CAUTI. |
|  | February & Holmes, 2020 | The Business Case for Magnet® in an International Hospital |  |  |
| Fewer central line-associated bloodstream infections (CLABSI) | Barnes et al., 2016 | Magnet® Hospital Recognition Linked to Lower Central Line-Associated Bloodstream Infection Rates | - | Revenues from the reduction of CLABSI. |
|  | Drenkard, 2022 | The Business Case for Magnet® Designation: Using Data to Support Strategy |  |  |
|  | February & Holmes, 2020 | The Business Case for Magnet® in an International Hospital |  |  |
|  | Rodríguez-García et al., 2020 | How Magnet Hospital Status Affects Nurses, Patients, and Organizations: A Systematic Review |  |  |
| Fewer  methicillin-resistant Staphylococcus aureus bloodstream infections (MRSA BSI) | Rodríguez-García et al., 2020 | How Magnet Hospital Status Affects Nurses, Patients, and Organizations: A Systematic Review | - | Revenues from the reduction of MRSA BSI. |
| Lower mortality rate | Connor et al., 2023 | The Association Between Magnet® Recognition and Patient Outcomes : A Scoping Review | - | Revenues from the reduction of the mortality rate. |
|  | Drenkard, 2022 | The Business Case for Magnet® Designation: Using Data to Support Strategy |  |  |
|  | McHugh et al., 2013 | Lower mortality in Magnet® hospitals |  |  |
|  | Petit Dit Dariel & Regnaux, 2015 | Do Magnet®-accredited hospitals show improvements in nurse and patient outcomes compared to non-Magnet hospitals: a systematic review |  |  |
|  | Rodríguez-García et al., 2020 | How Magnet Hospital Status Affects Nurses, Patients, and Organizations: A Systematic Review |  |  |
| Lower 30-day readmission rates | Drenkard, 2022 | The Business Case for Magnet® Designation: Using Data to Support Strategy | - | Revenues from the reduction of the 30-day readmission rate. |
| **Nursing personnel indicators** |  |  |  |  |
| Lower nurse turnover rates | Aiken et al., 2009 | The Magnet Nursing Services Recognition Program: a comparison of two groups of magnet hospitals | x | Revenue from the reduction of nurse turnover rates and the associated cost savings, both pre- and post-hire. |
|  | Drenkard, 2010 | The Business Case for Magnet® |  |  |
|  | Drenkard, 2022 | The Business Case for Magnet® Designation: Using Data to Support Strategy |  |  |
|  | February & Holmes, 2020 | The Business Case for Magnet® in an International Hospital |  |  |
|  | Higdon et al., 2013 | Business case for Magnet® in a small hospital |  |  |
|  | Kelly et al., 2012 | Nurse outcomes in Magnet® and non-magnet hospitals |  |  |
|  | Petit Dit Dariel & Regnaux, 2015 | Do Magnet®-accredited hospitals show improvements in nurse and patient outcomes compared to non-Magnet hospitals: a systematic review |  |  |
|  | Rodríguez-García et al., 2020 | How Magnet Hospital Status Affects Nurses, Patients, and Organizations: A Systematic Review |  |  |
| Lower use of agency nurses | Drenkard, 2010 | The Business Case for Magnet® | (x) | Revenue from the reduction of use of agency nurses. In case of ward closures, these reductions in agency use should be excluded. |
|  | Drenkard, 2022 | The Business Case for Magnet® Designation: Using Data to Support Strategy |  |  |
| Fewer sick days | Aiken et al., 2009 | The Magnet Nursing Services Recognition Program: a comparison of two groups of magnet hospitals | x | Revenue from the reduction of health-related absences of nurses. |
|  | Kelly et al., 2012 | Nurse outcomes in Magnet® and non-magnet hospitals |  |  |
|  | Petit Dit Dariel & Regnaux, 2015 | Do Magnet®-accredited hospitals show improvements in nurse and patient outcomes compared to non-Magnet hospitals: a systematic review |  |  |
|  | Rodríguez-García et al., 2020 | How Magnet Hospital Status Affects Nurses, Patients, and Organizations: A Systematic Review |  |  |
| Lower number of needlestick injuries | Higdon et al., 2013 | Business case for Magnet® in a small hospital | - | Revenue from the reduction of needlestick injuries |
|  | Stone & Gershon, 2006 | Nurse Work Environments and Occupational Safety in Intensive Care Units |  |  |
| **Hospital performance** |  |  |  |  |
| Extension of nursing services | - |  | x | Revenue from increase in patient volume, due to the extension of nursing services offered in the hospital. |
| Reduction in the length of stay | - |  | (x) | Revenue from the reduction in the length of stay of patients after their treatment. |
| Nurse-led innovations | Drenkard, 2022 | The Business Case for Magnet® Designation: Using Data to Support Strategy | x | Cost-savings from the implementation of nurse-led innovations. |
|  | February & Holmes, 2020 | The Business Case for Magnet® in an International Hospital |  |  |
